# Supplementary material for: Time-resolved cryo-EM visualizes ribosomal translocation with EF-G and GTP
Source: Nat Commun. 2021 Dec 13;12:7236. doi: 10.1038/s41467-021-27415-0 (PMC8668904; doi:10.1038/s41467-021-27415-0)
Supplement: Supplementary file 1 — Supplementary Information [file 41467_2021_27415_MOESM1_ESM.pdf]

Supplementary Discussion

Large-scale interdomain rearrangements of EF-G are unlikely to drive translocation

Mechanistic models involving large-scale interdomain EF-G dynamics of up to 100 Å partly rely on the crystallographic structure that reports a 70S ribosome with a highly compact EF-G•GDP [17]. Domain 4 in that structure is shifted by nearly 100 Å from its position in our structures and in numerous ribosome structures that capture extended EF-G in the presence of inhibitors (Supplementary Fig. 7). The compact EF-G was stalled on a pre-translocation-like non-rotated ribosome (similar to Structure I), using a non-productive aminoacyl-tRNA analog and EF-G mutant fused to ribosomal protein L9 of the crystallographically-related neighboring ribosome [17]. Extensive classification of non-rotated ribosomes in our 25-s dataset, including masks focused on the compact EF-G conformation, has not revealed a similar state. Although a minor population of ribosomes with compact EF-G might be below the limit of detection in our data, we note the compact crystallographic conformation appears incompatible with binding of EF-G with GTP. Indeed, formation of the 70S complex with L9-EF-G was only possible in the presence of GDP, whereas GTP prevented L9-EF-G from binding the ribosome [17]. In both free and ribosome-bound EF-G or EF-G homologs, domain 3 stabilizes sw-I in the presence of GTP or GTP analogs [16,18,51]. This GTP-compatible conformation sharply contrasts the compact L9-EF-G [17], in which domain 3 is shifted by 35 Å away from the GTPase center (Supplementary Fig. 7d). Furthermore, the compact EF-G places its GTPase domain ~4 Å away from its catalytic position near the SRL, incompatible with GTP hydrolysis. Finally, the area of contact between compact EF-G and the ribosome (~2500 Å^2) is much lower than that in Structure III (3730 Å^2). This indicates a less favorable affinity of compact EF-G to the non-rotated ribosome than that of extended EF-G to the rotated ribosome, in keeping with biochemical data [22]. Nevertheless, even if transient large-scale EF-G rearrangements occur on the ribosome [25], they must take place prior to formation of the pre-translocation Structure III and thus do not drive translocation.

Supplementary Table 1. Cryo-EM data collection and structure refinement statistics.

	I	II-A	II-B	III	III-vio	IV	V	VI	VII
**Data collection and processing**									
Magnification	105,000 x	105,000x	105,000x	105,000x	45,000x	105,000x	105,000x	105,000x	45,000x
Voltage (kV)	300	300	300	300	200	300	300	300	200
Electron exposure (e–/Å^2)	40.12	40.12; 40.21	40.12; 40.21	47.6	30.5	47.6	47.6	47.6	30.4
Defocus range (μm)	-0.5-1.5	-0.5-1.5	-0.5-1.5	-0.8-2.0	-0.5-1.5	-0.8-2.0	-0.8-2.0	-0.8-2.0	-0.5-1.5
Pixel size (Å)	0.83	0.83	0.83	0.8271	0.87	0.8271	0.8271	0.8271	0.87
Symmetry imposed	C1	C1	C1	C1	C1	C1	C1	C1	C1
Initial particle (no.)	137,421	686,850	686,850	475,746	517,847	475,746	475,746	475,746	170,799
Final particle (no.)	37,252	7,257	6,895	1,657	20,167	3,778	1,105	1,163	55,457
Map resolution (Å)**	3.0	3.2	3.2	3.8	3.2	3.3	3.9	3.8	2.9
FSC threshold	0.143	0.143	0.143	0.143	0.143	0.143	0.143	0.143	0.143
Map resolution range (Å)	2.5-4	2.5-4.5	2.5-4.5	3.0-6	3.0-4.5	3.0-6	3.0-6	3.0-6	2.5 -3.5
**Refinement**									
Initial model used (PDB code)	5UYM	5UYM	5UYM	5UYM	5UYM	5UYM	5UYM	5UYM	5UYM
Model resolution (Å)	3.0	3.2	3.2	3.8	3.2	3.3	3.9	3.8	2.9
Correlation Coefficient (cc_mask)*	0.84	0.84	0.85	0.72	0.82	0.75	0.71	0.71	0.90
Real space R-factor †	0.20	0.19	0.19	0.23	0.21	0.25	0.28	0.28	0.16
Map sharpening *B* factor (Å^2)	0	0	0	-65	0	-50	-50	-50	0
Model composition*									
Non-hydrogen atoms	147,982	148,980	145,452	153,376	154,413	153,154	149,827	147,614	149,037
Protein residues	5,721	6,070	5,604	6,630	6,761	6,599	6,173	5,887	6,084
RNA residues	4,811	4,735	4,735	4,735	4,735	4,736	4,736	4,736	4,732
B factors (Å^2)*									
Protein	134.17	131.99	129.90	144.31	156.77	107.03	134.96	125.75	138.75
RNA	148.09	117.33	128.68	134.42	136.93	103.33	134.05	124.82	130.50
R.m.s. deviations*,§									
Bond lengths (Å)	0.011	0.010	0.008	0.009	0.007	0.010	0.010	0.010	0.012
Bond angles (°)	1.1	1.1	1.0	1.0	1.0	1.1	1.1	1.1	1.1
Validation*									
MolProbity score	1.87	2.31	1.89	2.24	2.13	2.22	2.46	2.38	1.74
Clashscore	6.15	15.58	5.92	13.32	9.68	13.85	14.11	14.79	4.74
Poor rotamers (%)	0.71	0.89	0.90	1.07	1.01	1.0	1.63	0.85	0.73
Ramachandran plot*									
Favored (%)	90.71	87.36	89.22	88.68	87.39	89.83	86.08	82.16	91.82
Allowed (%)	9.29	12.27	10.41	10.89	11.32	9.88	13.56	17.84	8.18
Disallowed (%)	0.00	0.37	0.37	0.43	1.29	0.29	0.36	0.00	0.00
Validation (RNA)*									
Good sugar pucker (%)	99.6	99.5	99.7	99.8	99.6	99.5	99.8	99.8	99.4
Good backbone (%)#	78.5	82.3	78.8	82.0	83.5	80.8	76.6	77.4	78.6

** from Frealign (FSC_part)
* from Phenix
† from RSRef
§ root mean square deviations
# RNA backbone suites that fall into recognized rotamer conformations defined by Molprobity

Supplementary Table 2. Conformations of the 30S subunit (body rotation and head swivel) and EF-G in 70S translocation structures with 2 tRNAs and EF-G from this work and previous studies with antibiotics, EF-G mutations or GTP analogs.

**Structure** (method; reference; PDB code)	**Formed with EF-G and:**	[#] **Body rotation;** **Head swivel (°)**	**tRNA conformations**	**EF-G conformation** (GTP, GTPase center)
**Structures I – VII (this work; cryo-EM)**				
I	**GTP**	0.6; 0.7	A/A and P/P	No EF-G
II-A		10.7; 2.9	A/A and P/E	No EF-G
II-B		11.0; 3.7	A/P* and P/E	No EF-G
III		11.6; 3.5	A/P* and P/E	Ordered switch loops, **GDP and Pi** resolved (or, less likely GTP) near SRL
IV		5.0; 17.0	ap/P and pe/E	Disordered switch loops, **GDP near SRL** (slightly shifted away, relative to Structure III)
V		1.1; 18.1	ap*/P and pe*/E	Density for GTPase domain 1 and domain 2 is absent, domain 3 weak.
VI		1.1; 18.9	ap*/P and pe*/E	No EF-G
VII		0; 0	P/P and E/E	No EF-G
**Structures of 70S•2tRNA•EF-G complexes captured with inhibitors and/or mutations**				
**Pre-translocation-like:**				
7.6 Å cryo-EM, *E. coli*; Brilot et al, 2013; PDB 4V7D	Vio, Fus, and **GTP**	12.1; 3.4	A/P* and P/E	Ordered GTPase near SRL; insufficient resolution.
3.2 Å cryo-EM, *E. coli* (**this work**)	Vio and **GTP**	12.2; 3.4	A/P* and P/E	Ordered switch loops, **GDP and Pi**
2.8 Å X-ray; *T. thermophilus*; Lin et al, 2015; PDB 4WPO	L9-EF-G fusion mutant, non-hydrolyzable aminoacyl-tRNA analogs and **GDP**	2.3; 1.5	A/A and P/P	Compact EF-G, domain 3 shifted away from GTPase; GTPase ~10 Å from SRL; switch loops disordered; **GDP**
**Mid-translocation-like (swiveled head)**				

2.5 Å cryo-EM, E. coli; Rundlet et al, 2021; PDB 7N2V	Spc and GTP	10.9; 6.1	A/P* and P/E	Ordered GTPase near SRL; GDP•Pi density (reported as GTP or GTP/GDP•Pi mixture)
6.8 Å cryo-EM, *E. coli*; Ramrath et al. 2013; PDB 4V7B	Fus and **GTP**	5.1; 16.4	ap/P and pe/E	Disordered switch loops
3.8 Å X-ray, *T. thermophilus*; Zhou et al, 2014; PDB 4W29	Fus, Neomycin, and **GTP**	4.6; 18.7	ap/ap and pe/E	Disordered switch loops, Fus, **GDP**
3.5 Å cryo-EM, *E. coli;* (Structure II in Demo et al, 2021); PDB 7K51	**GDPCP**	5.7; 15.8	ap/P and pe/E	Ordered switch loops, **GDPCP**
**Post-translocation-like (nearly non-rotated)**				
3.6 Å cryo-EM; *E. coli*; Li et al, 2015; PDB 3J9Z	H91A EF-G mutant and **GTP**	0.4; 1.0	P/P and E/E	Ordered switch, GTP
3.6 Å X-ray; *T. thermophilus*; Gao et al, 2009; PDB 4V5F	Fus and **GDP**	2.6; 1.3	P/P and E/E	Disordered switch loops, Fus, **GDP**
3.4 Å cryo-EM, *E. coli;* (Structure III in Demo et al, 2021); PDB 7K52	**GDPCP**	2.5; 2.1	P/P and E/E	Ordered switch loops, **GDPCP**

[#] 30S body rotation and head swivel are measured relative to the non-rotated post-translocation Structure VII.

Supplementary Table 3. Statistics of local real-space refinement of GTP or GDP•Pi structural models into the maps corresponding to early EF-G intermediates.

	Correlation Coefficient (start)	Deviation from ideal stereochemistry				
		RMS angle (start)	RMS bond (start)	Chirality (start)	Planarity (start)	Dihedral angles (start)
**Map: III$_{gtpase}$**						
GDP•Pi (III)	0.56 (0.53)	1.66 (2.21)	0.005 (0.009)	0.075 (0.077)	0.000 (0.003)	15.36 (40.42)
GDP•Pi (4RD1)	0.56 (0.57)	1.67 (2.14)	0.005 (0.006)	0.075 (0.082)	0.000 (0.002)	14.58 (47.85)
GTP (4RD1)	0.42 (0.57)	1.64 (2.88)	0.003 (0.008)	0.081 (0.134)	0.003 (0.005)	30.04 (94.54)
**Map: III-vio**						
GDP•Pi (III-vio)	0.68 (0.72)	1.68 (2.00)	0.005 (0.010)	0.069 (0.090)	0.001 (0.003)	10.13 (50.08)
GDP•Pi (4RD1)	0.68 (0.57)	1.69 (2.14)	0.005 (0.006)	0.072 (0.083)	0.000 (0.002)	10.33 (47.85)
GTP (4RD1)	0.43 (0.48)	1.65 (2.88)	0.003 (0.008)	0.079 (0.133)	0.003 (0.005)	27.37 (94.53)
**Map: EMD-24134**						
GDP•Pi (4RD1)	0.68 (0.70)	1.74 (2.14)	0.006 (0.006)	0.076 (0.083)	0.000 (0.002)	13.80 (47.86)
GTP (4RD1)	0.58 (0.68)	1.61 (2.87)	0.003 (0.008)	0.079 (0.135)	0.004 (0.005)	34.68 (94.55)
GTP (Rundlet)	0.58 (0.70)	1.60 (6.33)	0.003 (0.034)	0.079 (0.074)	0.004 (0.001)	34.62 (91.41)

Supplementary Figures

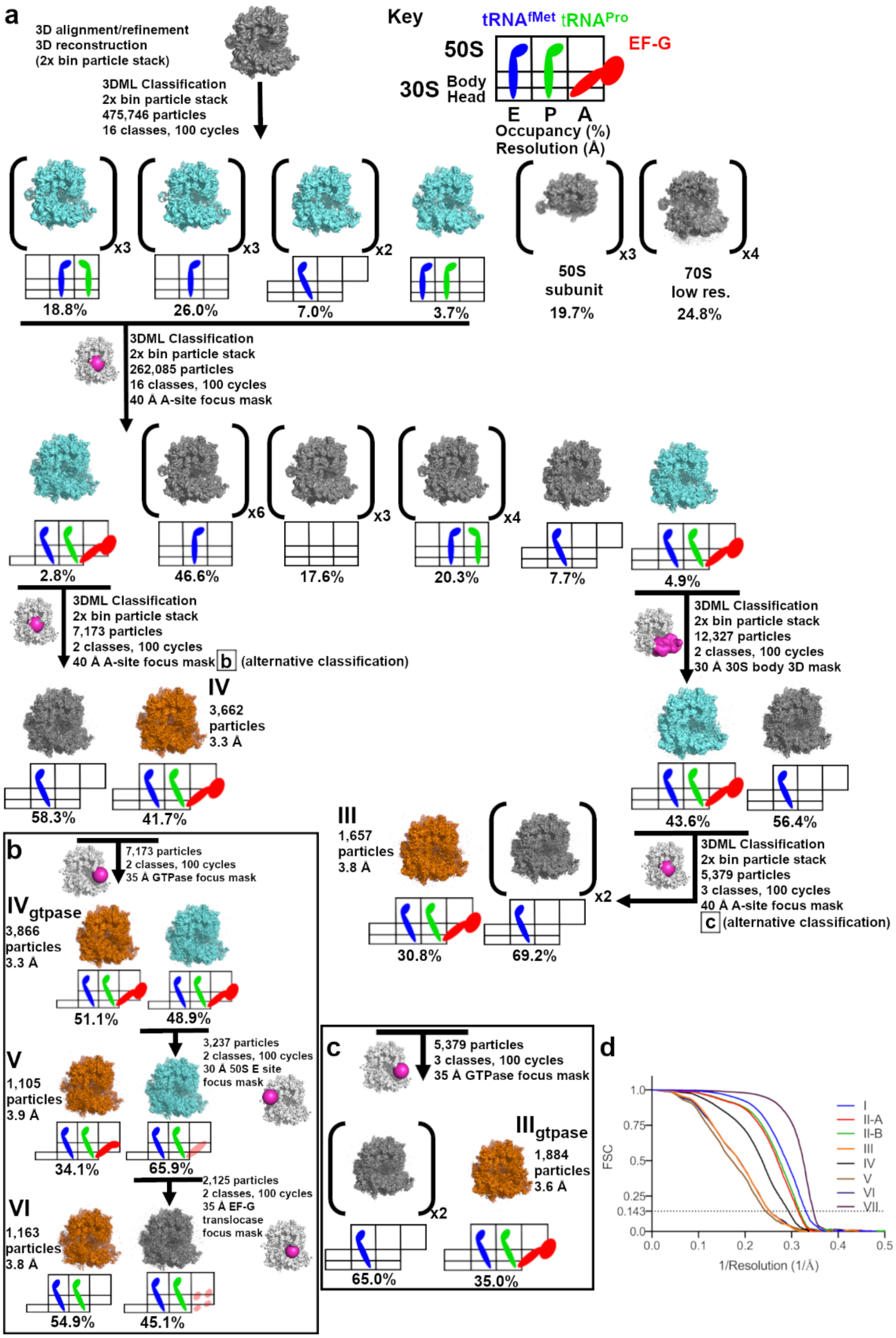

Supplementary Fig. 1. Scheme of maximum-likelihood classification of the 25-second data set resulting in Structures III to VI. a., Classification scheme and key showing tRNA and EF-G binding sites on the ribosome. **b-c.,** Alternative classification schemes. **d.,** FSC between even- and odd-particle half maps for each Structure in 0-s, 25-s, and 3600-s data sets.

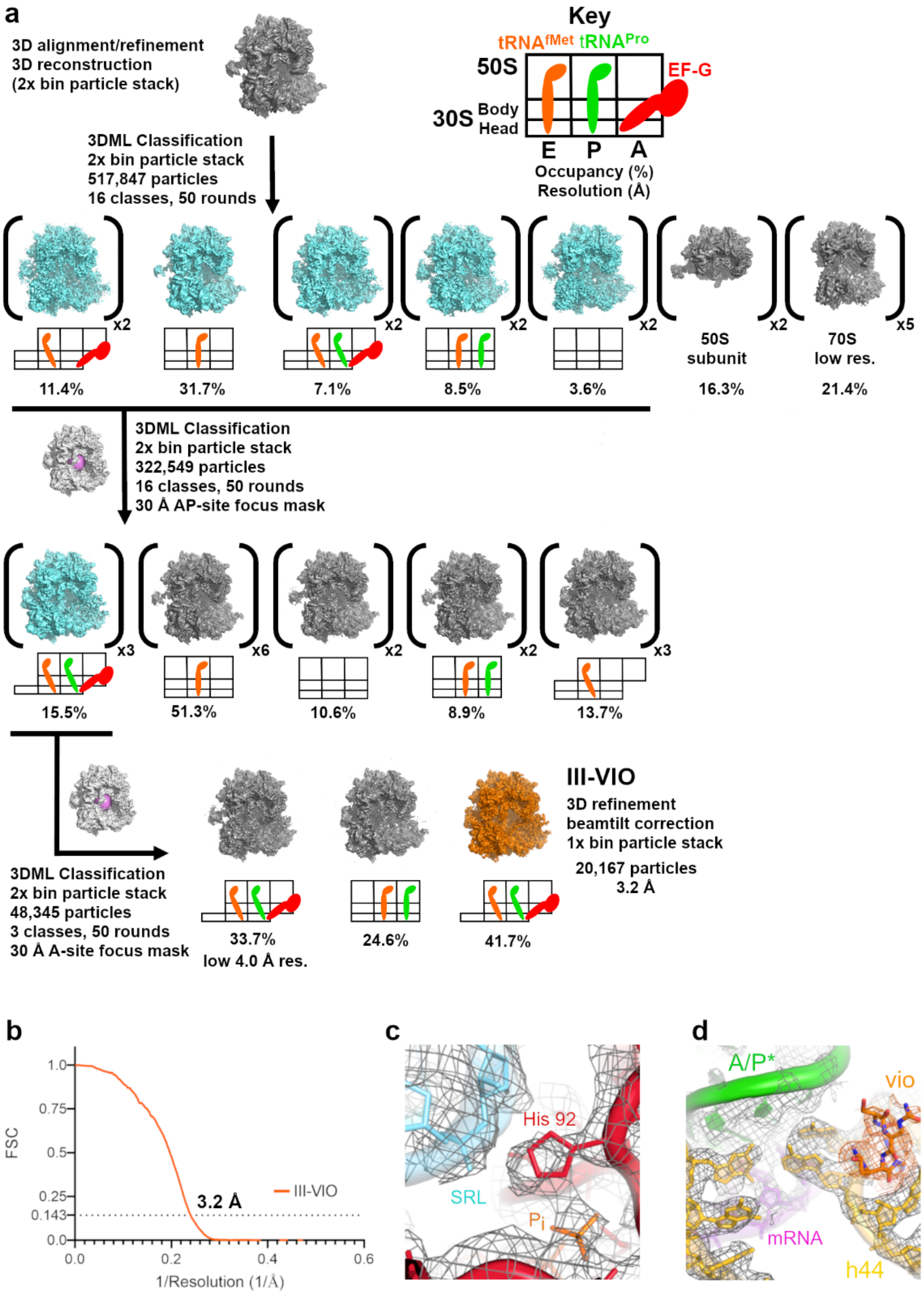

Supplementary Fig. 2. Scheme of maximum-likelihood classification of the data set resulting in Structure III-vio. a., Classification scheme and key showing tRNA and EF-G binding sites on the ribosome. **b.,** FSC between even- and odd-particle half maps for Structure III-vio. **c-d.,** Close-up views of cryo-EM density (mesh) for the GTPase center of EF-G near SRL (c) and the decoding center of the 30S subunit bound with viomycin (d).

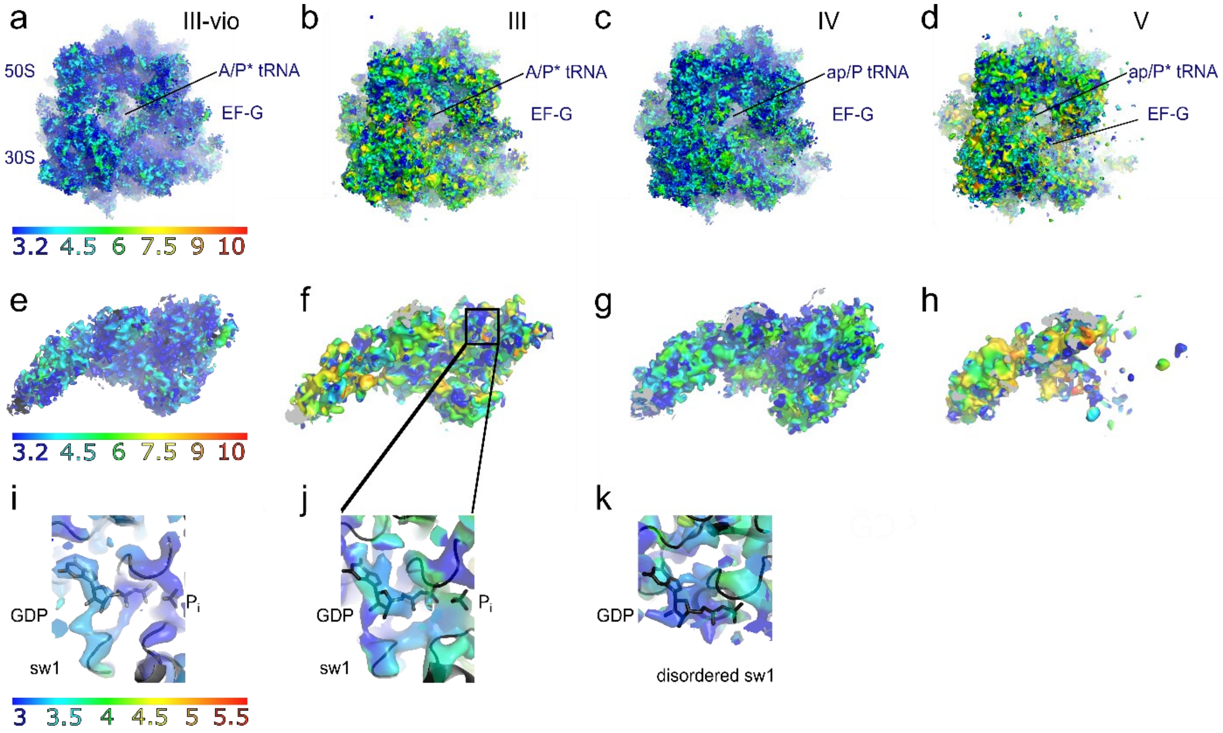

Supplementary Fig. 3. **Local resolutions for the EF-G-bound Structures III-vio, III, IV and V). a.,** An overview of the Structure III-vio map. The sharpened map (B-factor= -50) is coloured using a local-resolution scale ranging from 3.2 Å to 10 Å. **b-d.,** Overviews of Structures III through V. The sharpened maps (III B-factor = -65; IV B-factor = -50; V B-factor = -50) are coloured using a local-resolution scale ranging from 3.2 Å to 10 Å. **e-h.,** Local resolution of EF-G density in Structures III-vio, III, IV and V. **i-k**., Close-up views of local resolutions of the GTPase center of EF-G in Structures III-vio, IV and V. Local resolution of the maps was assessed using blocres and blocfilt from the Bsoft package, as described in Methods.

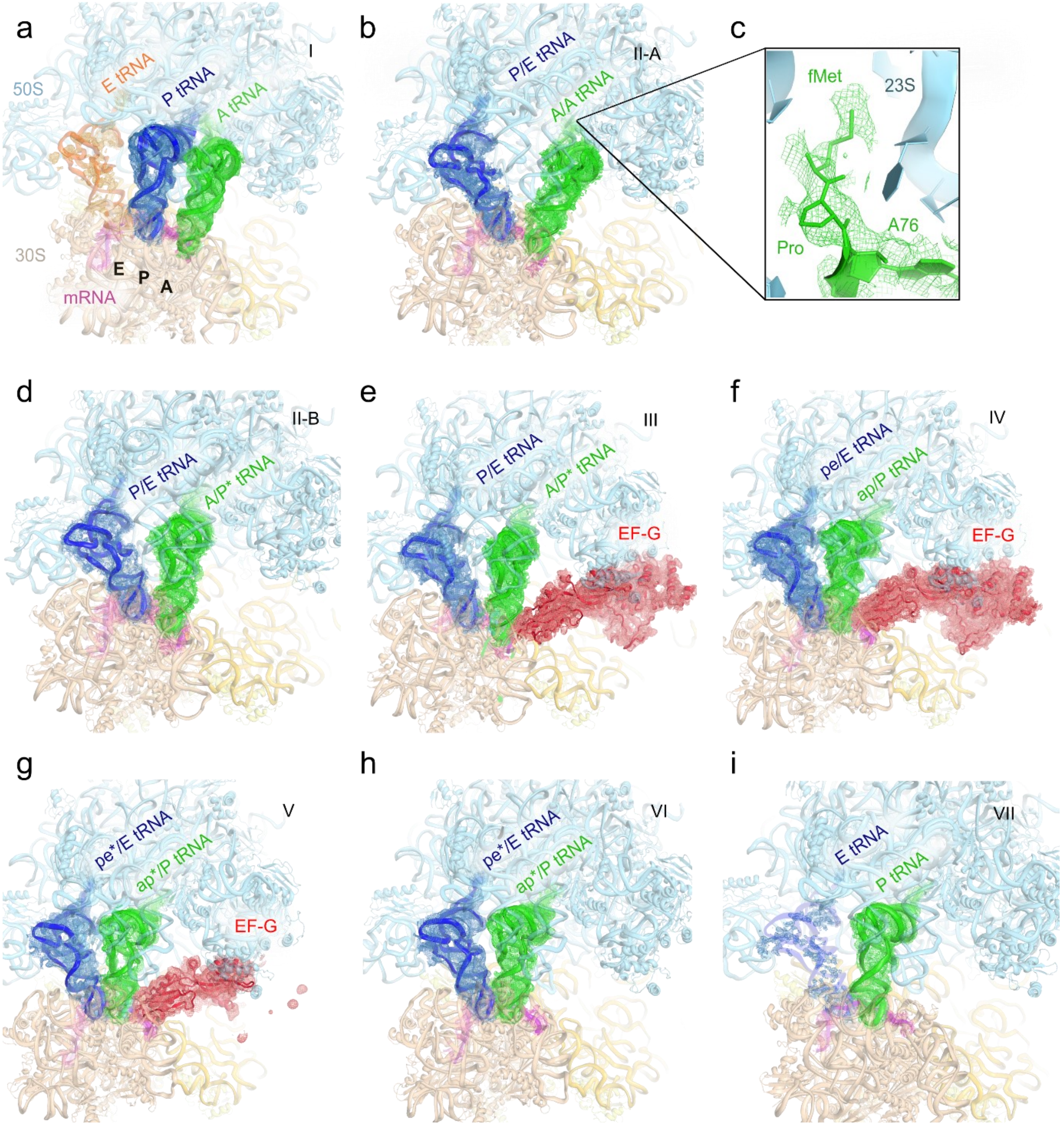

Supplementary Fig. 4. **Cryo-EM densities for tRNAs and EF-G in Structures I through VII.** All tRNA and EF-G densities are set to the same sigma within each panel. **a.,** Cryo-EM densities (mesh) for A-tRNA (green), P-tRNA (blue), E-tRNA (weak density; orange) and mRNA (magenta) in Structure I, shown with the 70S structural model, colored as in Figure 1. **b.,** Cryo-EM densities for A/A tRNA (green), P/E tRNA (blue) and mRNA (magenta) in Structure II-A. **c.,** Close-up view of dipeptidyl-tRNA density (fMet-Pro-tRNA^{Pro}) for the A/A tRNA (green) in the peptidyl transferase center of Structure II-A. **d.,** Cryo-EM densities for A/P* tRNA (green), P/E tRNA (blue) and mRNA (magenta) in Structure II-B. **e.,** Cryo-EM densities for A/P* tRNA (green), P/E tRNA (blue), mRNA (magenta) and EF-G (red) in Structure III. **f.,** Cryo-EM densities for ap/P tRNA (green), pe/E tRNA (blue), mRNA (magenta) and EF-G (red) in

Structure IV. **g.,** Cryo-EM densities for ap*/P tRNA (green), pe/E tRNA (blue), mRNA (magenta) and EF-G (red) in Structure V. **h.,** Cryo-EM densities for ap*/P tRNA (green), pe/E tRNA (blue) and mRNA (magenta) in Structure VI. **i.,** Cryo-EM densities for P-tRNA (green), E-tRNA (weak density; blue) and mRNA (magenta) in Structure VII.

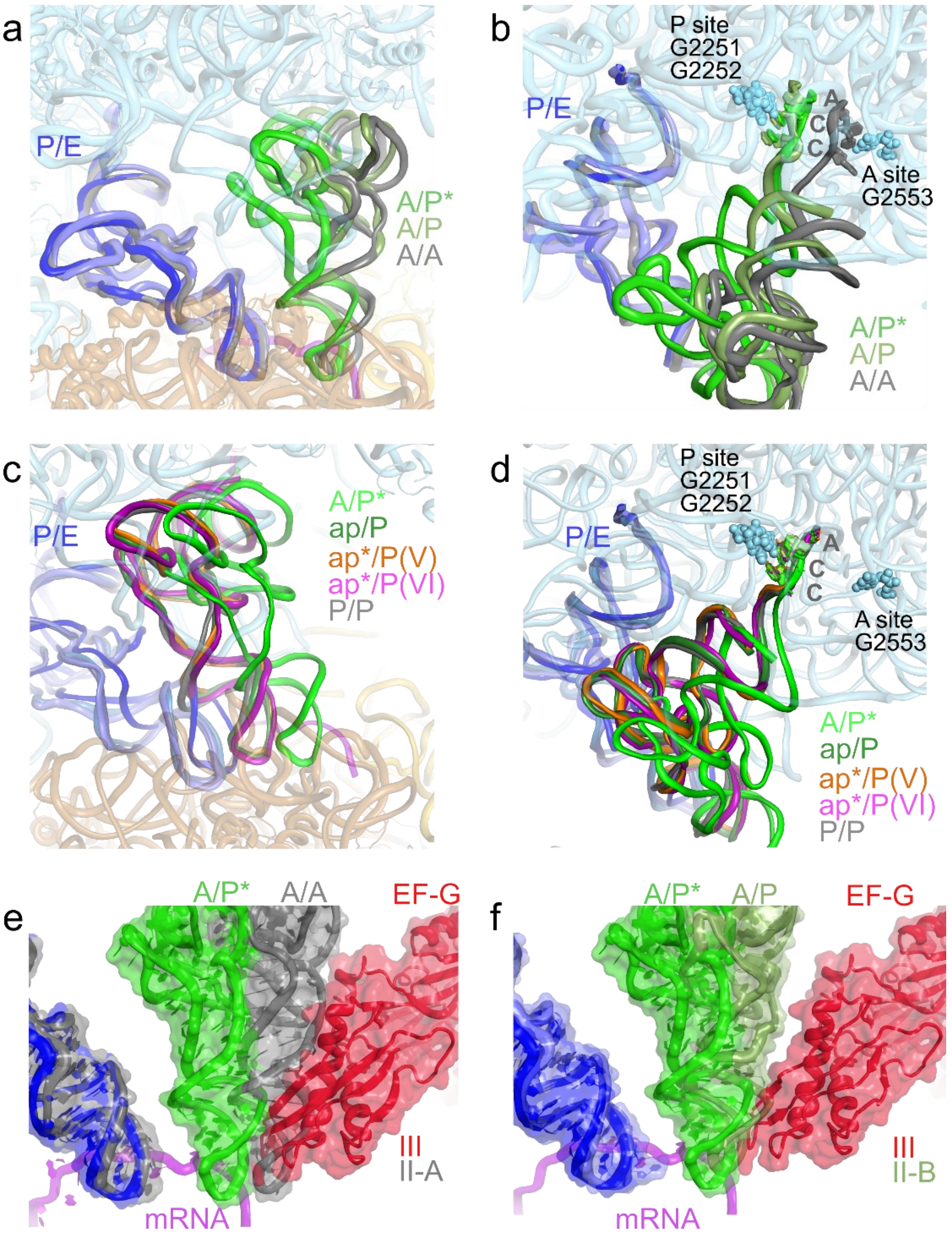

Supplementary Fig. 5. **Positions and conformations of A/A, A/P and A/P* tRNA in 30S-rotated Structures II-A, II-B, and II-C, respectively. a.,** Alignment of Structures II-A with A/A tRNA, PDB 6WDF with A/P tRNA, and II-B with A/P* tRNA show how the dipeptidyl-tRNA advances toward the 50S P site. P/E tRNA from all three structures (grey, slate, blue) is shown for reference. Structures were superimposed on 23S rRNA to compare tRNA positions relative to the 50S subunit (in panels a-d). **b.,** The CCA end of A/A tRNA resides in the 50S A site (G2553) while the CCA end of A/P (PDB 6WDF) and A/P* tRNA resides in the 50S P site (G2251-2252). **c.,** Alignment of Structures III, IV, V, VI, and VII show that $tRNA^{Pro}$ (green, forest, orange, purple, grey) occupies the 50S P site. P/E tRNA from all structures except VII (blue, deep blue, sky blue, slate) is shown for reference. **d.,** The CCA end of $tRNA^{Pro}$ resides in the 50S P site (G2251-2252) for all labeled structures. **e-f.,** Structural alignment of the 30S subunit

shows that EF-G domain 4 in Structure III would sterically clash with A/A (grey; Structure II-A) and A/P (smudge green; PDB 6WDF) tRNAs.

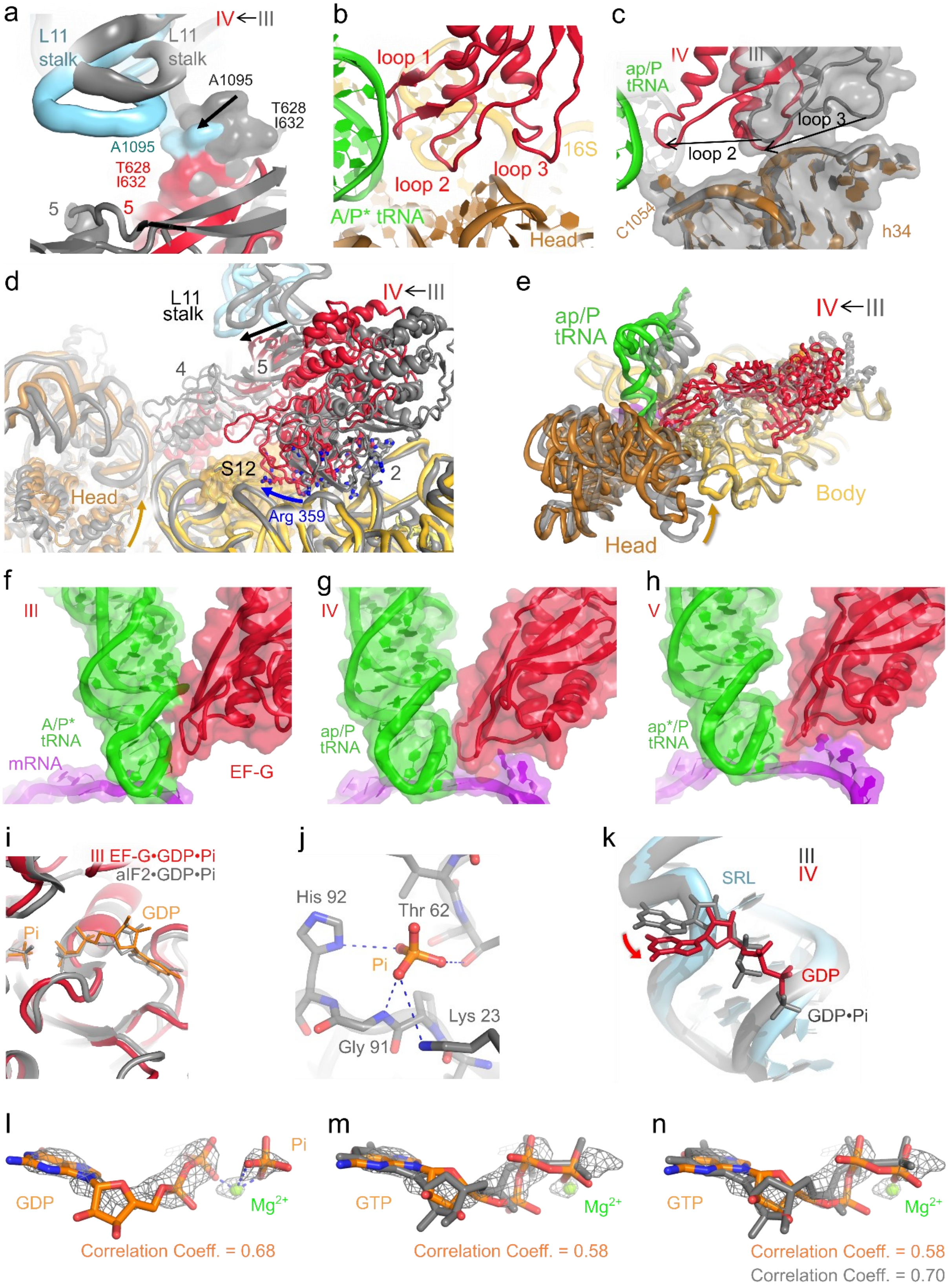

a
IV←III
L11 stalk
L11 stalk
A1095
T628 I632
A1095
T628 I632
5
5
b
loop 1
16S
loop 3
loop 2
A/P* tRNA
Head
c
ap/P tRNA
IV
III
loop 3
loop 2
C1054
h34
d
L11 stalk
IV←III
4
5
S12
2
Head
Arg 359
e
ap/P tRNA
IV←III
Body
Head
f
III
A/P* tRNA
mRNA
EF-G
g
IV
ap/P tRNA
h
V
ap*/P tRNA
i
III EF-G•GDP•Pi
aIF2•GDP•Pi
GDP
Pi
j
His 92
Thr 62
Pi
Lys 23
Gly 91
k
III
IV
SRL
GDP
GDP•Pi
l
Pi
GDP
Mg2+
Correlation Coeff. = 0.68
m
GTP
Mg2+
Correlation Coeff. = 0.58
n
GTP
Mg2+
Correlation Coeff. = 0.58
Correlation Coeff. = 0.70

Supplementary Fig. 6. **Comparison of EF-G in Structures III and IV. a.,** Alignment on 23S rRNA shows L11 residue 1095 packing on EF-G residues 628 and 632 in Structure III and IV. **b.,** Interactions of the three loops at the tip of EF-G domain 4 with the ribosomal rRNA and tRNA in Structure III **c.,** Loops 2 and 3 of EF-G domain 4 change positions to interact with the same region of the 30S head domain in Structures III and IV, respectively. **d.,** Positively charged residues of EF-G domains 2 and 3 (shown as sticks) slide along 16S rRNA toward S12, from Structure III to IV. **e.,** Comparison of the positions of the translocating dipeptidyl-tRNA, EF-G and 30S head in Structures III (gray) and IV (colored, as specified by the labels). Structures III and IV were superimposed by the structural alignment of the 30S body domain (all rRNA nucleotides except for 921-1397); ribosomal proteins are omitted for clarity. **f-h.,** EF-G directly contacts the ASL throughout translocation **i.,** Conformation of the GTPase center of EF-G in Structure III is similar to those in free translational GTPases bound with GTP: a crystal structure of an archaeal initiation factor aIF2•GTP is shown for comparison (gray; PDB 4RD1 [86]). **j.,** Coordination environment for the putative phosphate ion includes positively charged residues and hydrogen bonding donors (within 3.5 Å from the putative oxygen positions). **k.,** Alignment on 23S rRNA shows that GDP moves ~2 Å away from the SRL between structures III and IV. **l-n.**, Cryo-EM density from an early translocation intermediate EMD-24134 (Rundlet et al., 2021) is more consistent with GDP•Pi than GTP. GDP•Pi (l) and GTP (m) from the 1.5 Å crystal structure of an archaeal initiation factor aIF2-gamma H97A variant crystallized with GTP (PDB 4RD1) were refined into map EMD-24134 using phenix.real_space_refine to improve nucleotide stereochemical parameters (refined nucleotides=orange; fit nucleotides=grey; see Supplementary Table 3). Panel n shows the originally modeled GTP from corresponding PDB 7N2V.

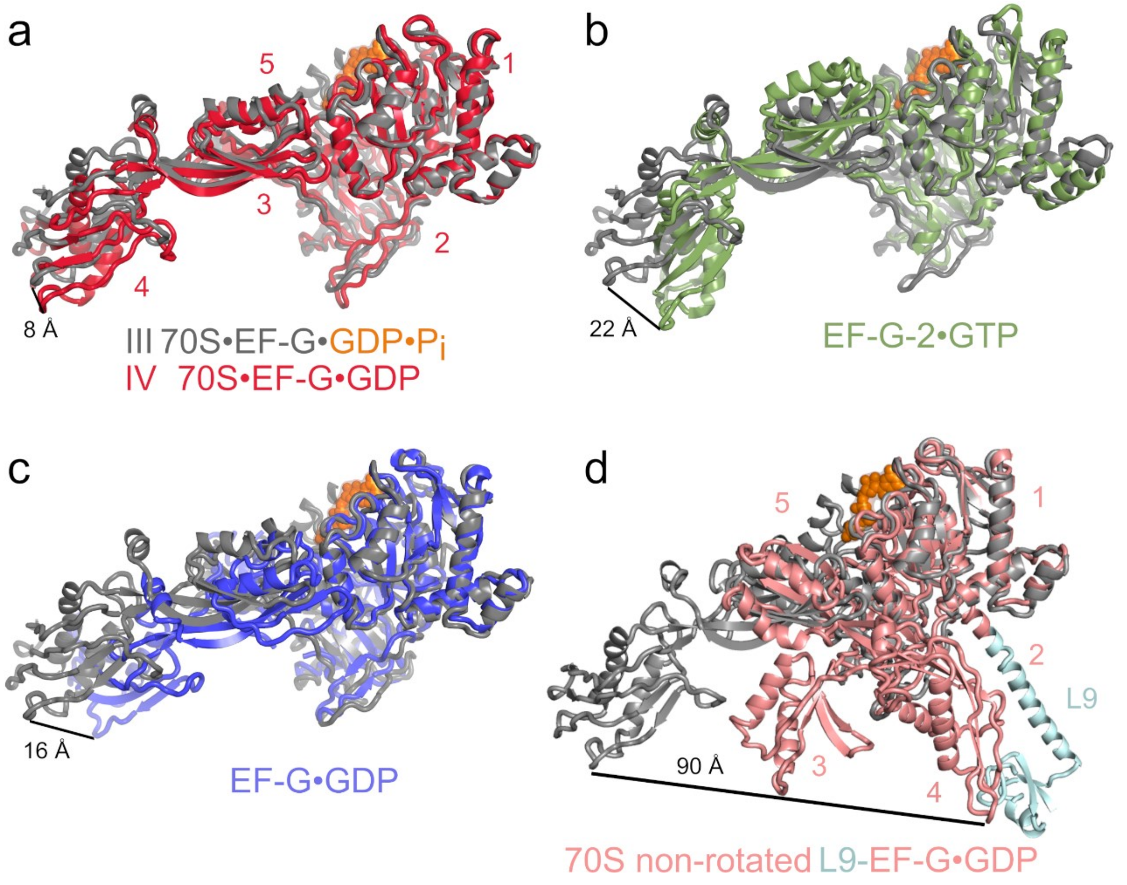

Supplementary Fig. 7. **Comparison of the position of EF-G domain 4 in the pre-translocation Structure III (gray with orange GDP*Pi) and in other structures of free and ribosome-bound EF-G and EF-G homologs. a.,** EF-G•GDP in Structure IV (red); **b.,** crystal structure of *Th. thermophilus* EF-G-2 with GTP (green; PDB 2DY1 [69]); **c.,** crystal structure of *Th. thermophilus* EF-G (blue; PDB 2EFG [62]); **d.,** compact *Th. thermophilus* L9-EF-G•GDP fusion protein crystallized in the non-rotated 70S ribosome (pink; PDB 4WPO [26]). Domains of EF-G (1-5) are labeled in panels a and d. Superposition was performed by structural alignment of domains 1-2s. The distance between the tips of domain 4 (at *E. coli* aa 511) is shown.

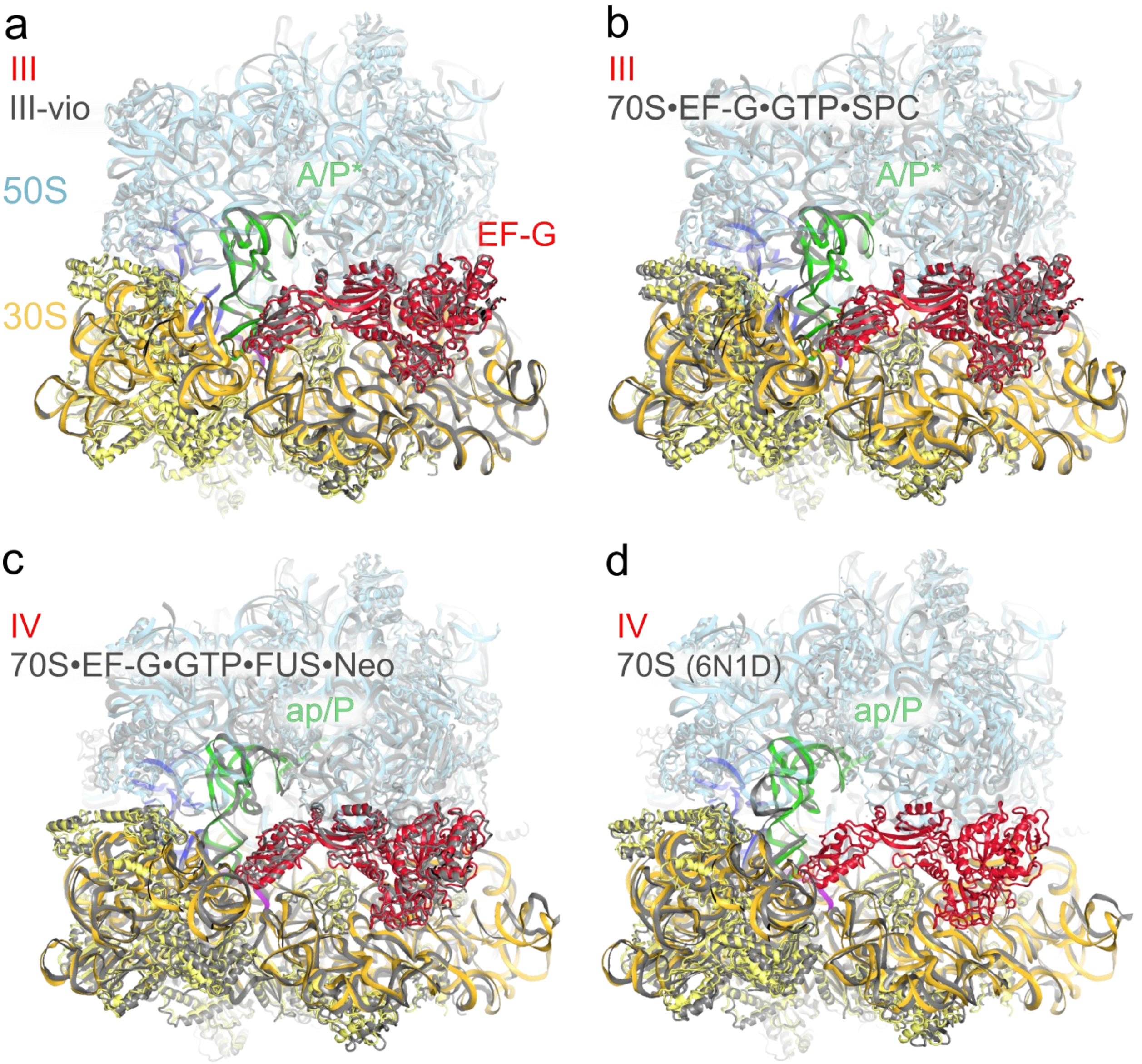

Supplementary Fig. 8. **Comparison of Structures III-V with previous structures determined using X-ray crystallography and/or inhibitors (aligned on 23S rRNA; see Supplementary Table 2 for quantitative comparisons). a.**, Structure III is nearly identical to III-vio (this work). **b.**, Structure III features a less rotated head and less advanced dipeptidyl-tRNA than an early EF-G-intermediate captured with spectinomycin using cryo-EM (PDB 7N2V; Rundlet et al, 2021). **c.**, Positions of EF-G, tRNAs and 30S subunit in Structure IV are similar to those in the crystal structure captured with fusidic acid and neomycin (PDB 4W29; Zhou et al, 2014). **d.**, Comparison of Structure IV with the crystal structure of a 70S•2tRNA complex formed without EF-G and featuring a highly swiveled head and destabilized codon-anticodon interactions (PDB 6N1D; Zhou et al, 2019). The extents of head swivel (17° in IV; 18.3° in 6N1D) and body rotation (5.0° in IV; 4.1° in 6N1D) in these structures are similar.
