# Supplementary material for: Time-resolved cryo-EM visualizes ribosomal translocation with EF-G and GTP
Source: Nat Commun. 2021 Dec 13;12:7236. doi: 10.1038/s41467-021-27415-0 (PMC8668904; doi:10.1038/s41467-021-27415-0)
Supplement: Supplementary file 3 — Description of Additional Supplementary Files [file 41467_2021_27415_MOESM3_ESM.pdf]

Description of Additional Supplementary Files

File Name: Supplementary Movie 1

Description: Animation showing tRNA and mRNA translocation by EFG•GTP. Two scenes demonstrate how translocation is coupled with conformational changes of the small 30S subunit: 1) 30S body rotation, and 2) 30S head swivel. Scene 3) shows a close-up view of EF-G GTPase center binding near the sarcin ricin loop (SRL). Structures I through VII are labeled. The GTPase superdomain of EF-G in Structure V (not resolved in cryo-EM data; shown in pink) is approximated by two models obtained by superposition with EF-G from Structure IV (first model) and from PDB ID 2EFG (second model).
