# Supplementary material for: Time-resolved cryo-EM visualizes ribosomal translocation with EF-G and GTP
Source: Nat Commun. 2021 Dec 13;12:7236. doi: 10.1038/s41467-021-27415-0 (PMC8668904; doi:10.1038/s41467-021-27415-0)
Supplement: Supplementary file 5 — Reporting Summary [file 41467_2021_27415_MOESM5_ESM.pdf]

nature research

Corresponding author(s): Andrei A. Korostelev

Last updated by author(s): Nov 12, 2021

Reporting Summary

Nature Research wishes to improve the reproducibility of the work that we publish. This form provides structure for consistency and transparency in reporting. For further information on Nature Research policies, see our Editorial Policies and the Editorial Policy Checklist.

Statistics

For all statistical analyses, confirm that the following items are present in the figure legend, table legend, main text, or Methods section.

n/a	Confirmed	
☒	☐	The exact sample size (*n*) for each experimental group/condition, given as a discrete number and unit of measurement
☒	☐	A statement on whether measurements were taken from distinct samples or whether the same sample was measured repeatedly
☒	☐	The statistical test(s) used AND whether they are one- or two-sided<br>*Only common tests should be described solely by name; describe more complex techniques in the Methods section.*
☒	☐	A description of all covariates tested
☒	☐	A description of any assumptions or corrections, such as tests of normality and adjustment for multiple comparisons
☒	☐	A full description of the statistical parameters including central tendency (e.g. means) or other basic estimates (e.g. regression coefficient) AND variation (e.g. standard deviation) or associated estimates of uncertainty (e.g. confidence intervals)
☒	☐	For null hypothesis testing, the test statistic (e.g. *F*, *t*, *r*) with confidence intervals, effect sizes, degrees of freedom and *P* value noted<br>*Give P values as exact values whenever suitable.*
☒	☐	For Bayesian analysis, information on the choice of priors and Markov chain Monte Carlo settings
☒	☐	For hierarchical and complex designs, identification of the appropriate level for tests and full reporting of outcomes
☒	☐	Estimates of effect sizes (e.g. Cohen's *d*, Pearson's *r*), indicating how they were calculated

Our web collection on statistics for biologists contains articles on many of the points above.

Software and code

Policy information about availability of computer code

Data collection	SerialEM (vs. 3.6), IMOD (vs. 4.9.0)
Data analysis	Graphpad Prism 8, cisTEM (version 1.0-beta and pre-release), Frealign v. 9.11 (Aug 2017 release; includes FrealignX), RSRef(2000), Phenix (1.14-3260) and Molprobity (1.14-3260), Chimera (vs. 1.13), PyMOL v1.7.x, IMOD (vs. 4.9.0), Bsoft v1.9.1

For manuscripts utilizing custom algorithms or software that are central to the research but not yet described in published literature, software must be made available to editors and reviewers. We strongly encourage code deposition in a community repository (e.g. GitHub). See the Nature Research guidelines for submitting code & software for further information.

Data

Policy information about availability of data

All manuscripts must include a data availability statement. This statement should provide the following information, where applicable:

- Accession codes, unique identifiers, or web links for publicly available datasets
- A list of figures that have associated raw data
- A description of any restrictions on data availability

The EM density maps generate in this study have been deposited in the EMDB under accession codes EMD-25420 (Structure I); EMD-25411 (Structure II-A); EMD-25410 (Structure II-B); EMD-25409 (Structure III); EMD-25407 (Structure IV); EMD-25405 (Structure V); EMD-25145 (Structure VI); EMD-25418 (Structure VII); EMD-25421 (Structure III-vio). The atomic coordinates generate in this study have been deposited in the PDB under accession codes 7ST6 (Structure I); 7SSO (Structure II-A); 7SSN (Structure II-B); 7SSL (Structure III); 7SSD (Structure IV); 7SS9 (Structure V); 7SSW (Structure VI); 7ST2 (Structure VII); 7ST7 (Structure III-vio). Structures from prior studies were used in this work for comparison and are available in the Protein Data Bank: 6WDF, 5U9F, 4V7D, 7K51, 4V9H, 4W29, 1WDT, 4RD1, 5UYM, 4WPO, 7N2V, 4V7B, 3J9Z, 4V5F, 7K52, 2DY1, and 2EFG. Additionally, the corresponding map for PDB 7N2V (EMD-24134) was used in refinement analyses (Methods).

Field-specific reporting

Please select the one below that is the best fit for your research. If you are not sure, read the appropriate sections before making your selection.

☒ Life sciences ☐ Behavioural & social sciences ☐ Ecological, evolutionary & environmental sciences

For a reference copy of the document with all sections, see nature.com/documents/nr-reporting-summary-flat.pdf

Life sciences study design

All studies must disclose on these points even when the disclosure is negative.

Sample size	Cryo-EM Datasets for each complex were collected such that a near-atomic resolution of ~3-4 A could be reached as has been determined previously (Loveland et al. Nature. 2020; Svidritskiy et al. eLife. 2019). Datasets of 100,000-200,000 particles were sufficient to reach this resolution for the 0 and 3600 second time points as determined from numerous previous studies. The larger dataset of ~500,000-700,000 was collected for the 0 and 25 second time point to increase the chances of capturing lowly populated intermediate states.
Data exclusions	No data was excluded.
Replication	Cryo-EM experiments were not repeated unless grid conditions needed further optimization as explained above.
Randomization	Computational approaches to unbiased particle classification (maximum likelihood classification) include randomizations. Classifications were repeated multiple times typically by varying number of classes or mask position; despite these changes, the classifications converged on similar states. In the case of the 0 second time point, two independent datasets were collected. Classification of these datasets revealed similar states.
Blinding	Cryo-EM data sets were collected and processed by different individuals. Analyses of both data sets were performed using the same approach so as to avoid biases. Further blinding is not required/feasible in cryo-EM data processing; however, multiple authors reviewed and interpreted maps similarly throughout data processing

Reporting for specific materials, systems and methods

We require information from authors about some types of materials, experimental systems and methods used in many studies. Here, indicate whether each material, system or method listed is relevant to your study. If you are not sure if a list item applies to your research, read the appropriate section before selecting a response.

Materials & experimental systems

n/a	Involved in the study
☒	☐ Antibodies
☒	☐ Eukaryotic cell lines
☒	☐ Palaeontology and archaeology
☒	☐ Animals and other organisms
☒	☐ Human research participants
☒	☐ Clinical data
☒	☐ Dual use research of concern

Methods

n/a	Involved in the study
☒	☐ ChIP-seq
☒	☐ Flow cytometry
☒	☐ MRI-based neuroimaging
